# Supplementary material for: Computer-Aided Histopathological Characterisation of Endometriosis Lesions
Source: J Pers Med. 2022 Sep 16;12(9):1519. doi: 10.3390/jpm12091519 (PMC9504345; doi:10.3390/jpm12091519)
Supplement: Supplementary file 1 [file jpm-12-01519-s001.zip › jpm-1890457 - supplementary.pdf]

Supplementary Figures and Tables

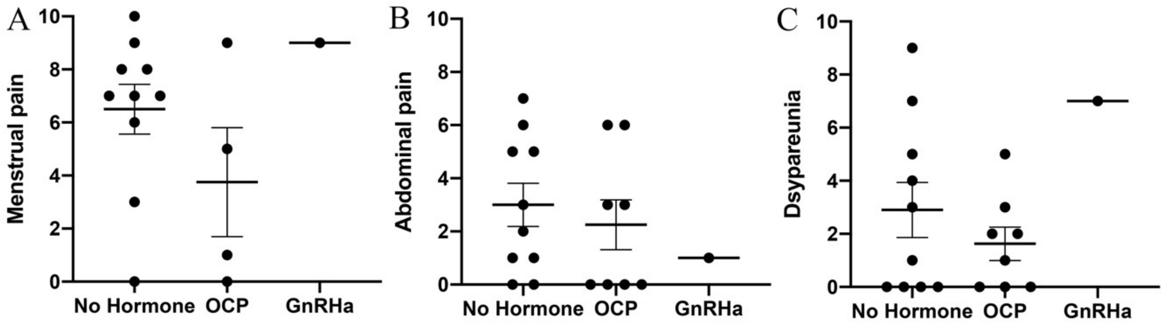

Supplementary Figure S1. Relationship between pain and hormonal treatment. Comparison of (**A**) menstrual pain, (**B**) abdominal pain, and (**C**) dyspareunia scores between samples derived from women who were not taking hormonal treatments compared to samples from women who were taking hormonal treatments split into oral contraceptive pill (OCP) and gonadotropin-releasing hormonal analogue (GnRHa), which showed a general decrease in pain compared to women who were not taking hormonal treatments (mo hormone), although the sample size was not sufficient for this to be accurately analysed statistically.

Supplementary Table S1. Parameters used by object classifier to identify cell type.

List of Parameters Used for Machine-Based Cell Detection	
1	Centroid X µm
2	Centroid Y µm
3	Nucleus: Area
4	Nucleus: Perimeter
5	Nucleus: Circularity
6	Nucleus: Max calliper
7	Nucleus: Min calliper
8	Nucleus: Eccentricity
9	Nucleus: Haematoxylin OD mean
10	Nucleus: Haematoxylin OD sum
11	Nucleus: Haematoxylin OD std dev
12	Nucleus: Haematoxylin OD max
13	Nucleus: Haematoxylin OD min
14	Nucleus: Haematoxylin OD range
15	Nucleus: DAB OD mean
16	Nucleus: DAB OD sum
17	Nucleus: DAB OD std dev
18	Nucleus: DAB OD max
19	Nucleus: DAB OD min
20	Nucleus: DAB OD range
21	Cell: Area
22	Cell: Perimeter
23	Cell: Circularity
24	Cell: Max calliper
25	Cell: Min calliper
26	Cell: Eccentricity

27	Cell: Haematoxylin OD mean
28	Cell: Haematoxylin OD std dev
29	Cell: Haematoxylin OD max
30	Cell: Haematoxylin OD min
31	Cell: DAB OD mean
32	Cell: DAB OD std dev
33	Cell: DAB OD max
34	Cell: DAB OD min
35	Cytoplasm: Haematoxylin OD mean
36	Cytoplasm: Haematoxylin OD std dev
37	Cytoplasm: Haematoxylin OD max
38	Cytoplasm: Haematoxylin OD min
39	Cytoplasm: DAB OD mean
40	Cytoplasm: DAB OD std dev
41	Cytoplasm: DAB OD max
42	Cytoplasm: DAB OD min
43	Nucleus/Cell area ratio
